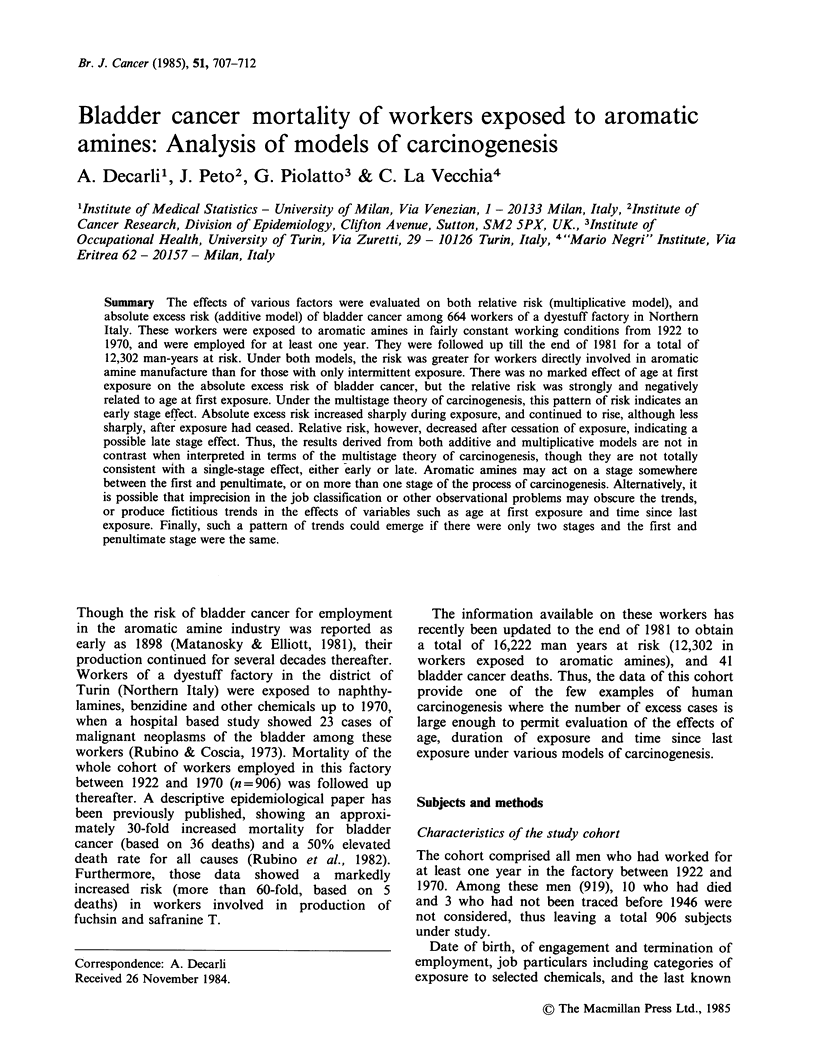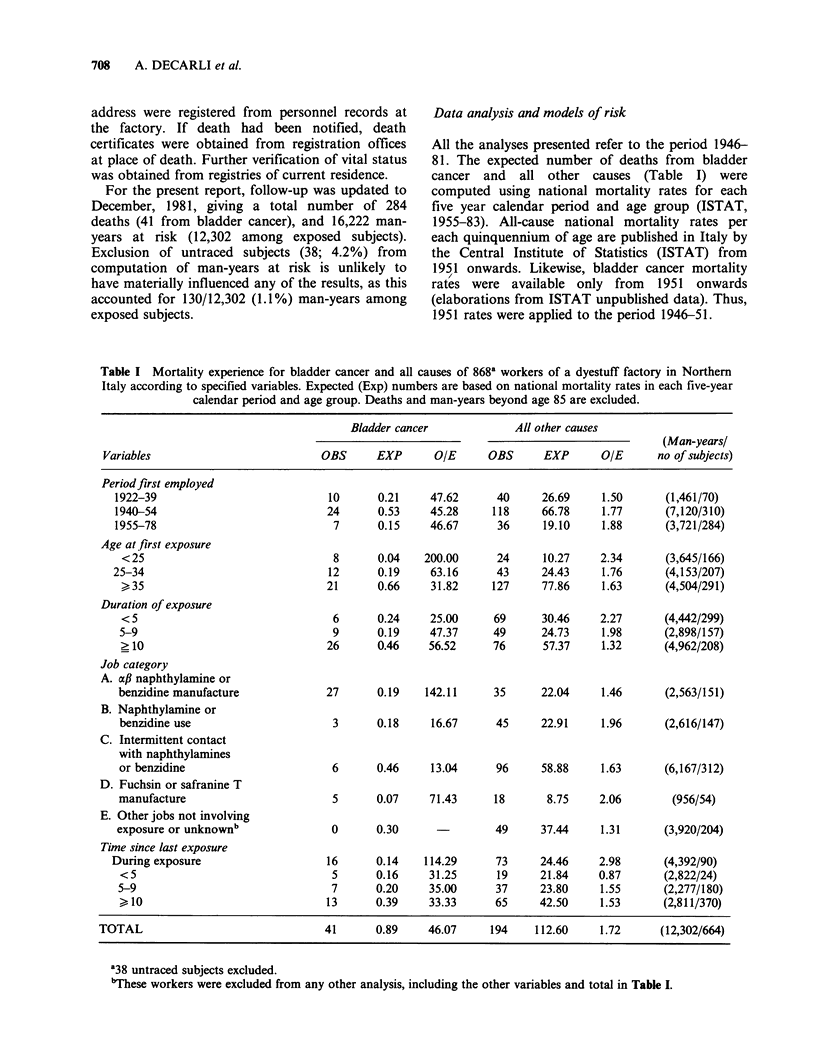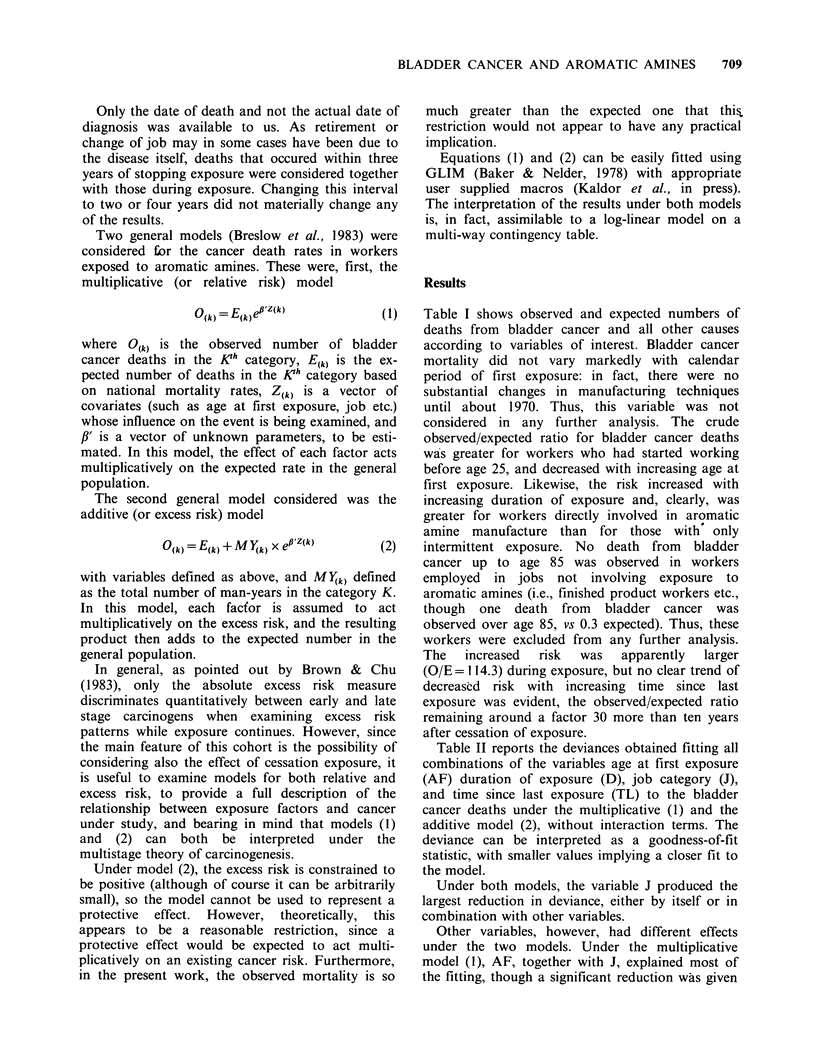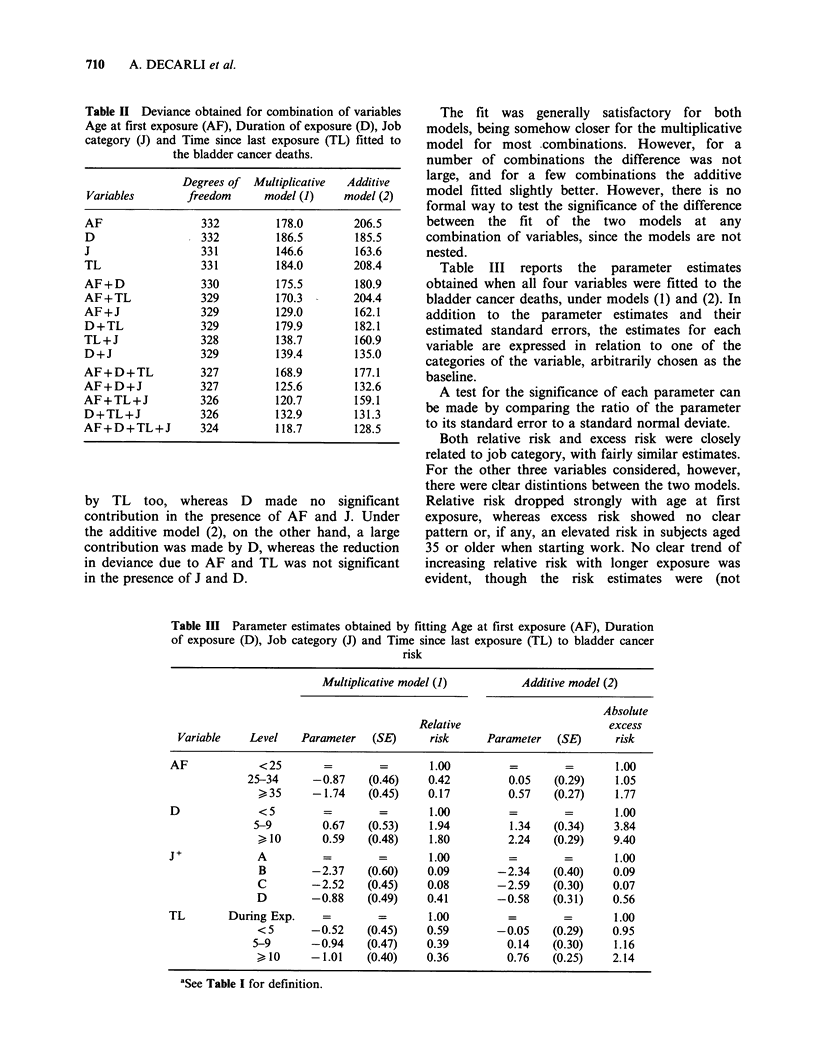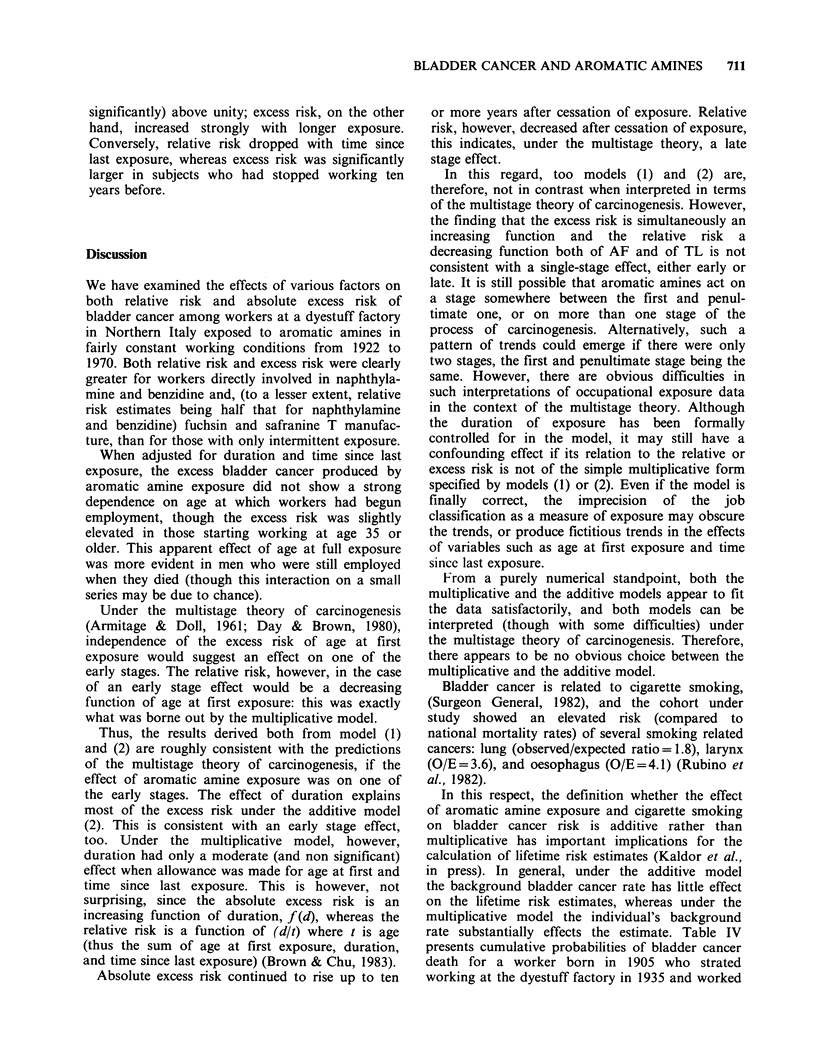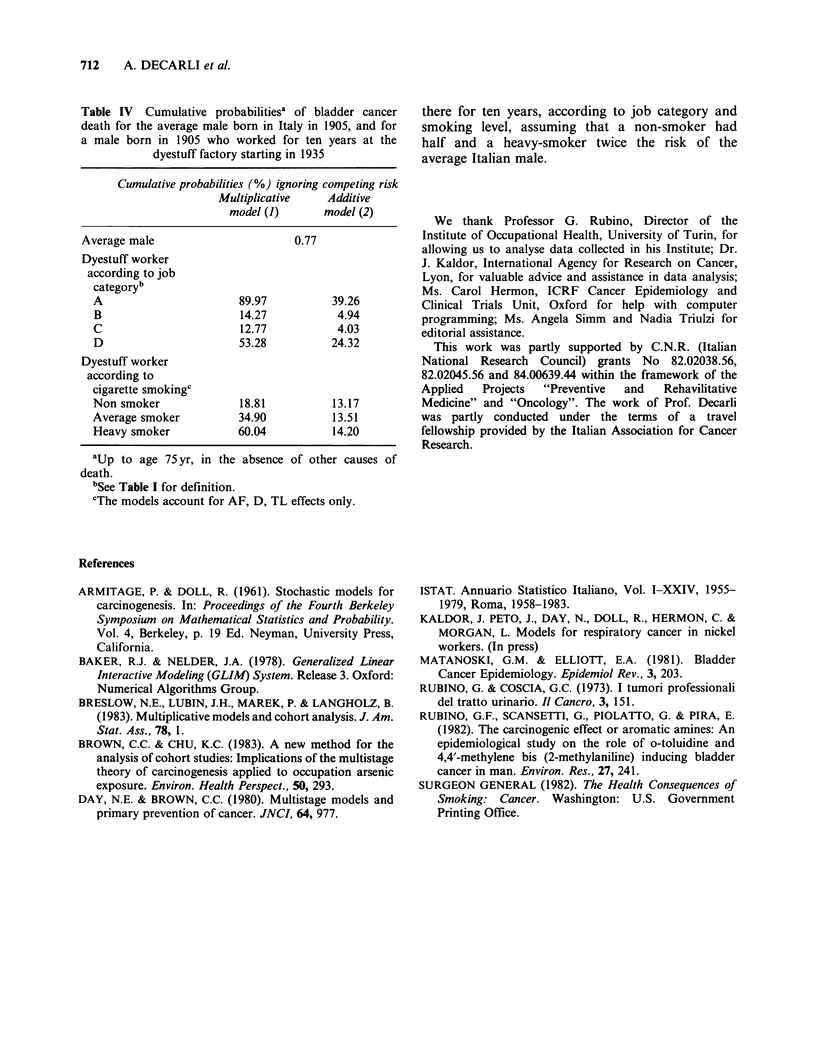# Bladder cancer mortality of workers exposed to aromatic amines: analysis of models of carcinogenesis.

**DOI:** 10.1038/bjc.1985.106

**Published:** 1985-05

**Authors:** A. Decarli, J. Peto, G. Piolatto, C. La Vecchia

## Abstract

The effects of various factors were evaluated on both relative risk (multiplicative model), and absolute excess risk (additive model) of bladder cancer among 664 workers of a dyestuff factory in Northern Italy. These workers were exposed to aromatic amines in fairly constant working conditions from 1922 to 1970, and were employed for at least one year. They were followed up till the end of 1981 for a total of 12,302 man-years at risk. Under both models, the risk was greater for workers directly involved in aromatic amine manufacture than for those with only intermittent exposure. There was no marked effect of age at first exposure on the absolute excess risk of bladder cancer, but the relative risk was strongly and negatively related to age at first exposure. Under the multistage theory of carcinogenesis, this pattern of risk indicates an early stage effect. Absolute excess risk increased sharply during exposure, and continued to rise, although less sharply, after exposure had ceased. Relative risk, however, decreased after cessation of exposure, indicating a possible late stage effect. Thus, the results derived from both additive and multiplicative models are not in contrast when interpreted in terms of the multistage theory of carcinogenesis, though they are not totally consistent with a single-stage effect, either early or late. Aromatic amines may act on a stage somewhere between the first and penultimate, or on more than one stage of the process of carcinogenesis. Alternatively, it is possible that imprecision in the job classification or other observational problems may obscure the trends, or produce fictitious trends in the effects of variables such as age at first exposure and time since last exposure. Finally, such a pattern of trends could emerge if there were only two stages and the first and penultimate stage were the same.


*Br. J. Cancer* (1985), **51**, 707–712

### Bladder cancer mortality of workers exposed to aromatic amines: Analysis of models of carcinogenesis

A. Decarli[1], J. Peto[2], G. Piolatto[3] & C. La Vecchia[4]

[1]*Institute of Medical Statistics – University of Milan, Via Venezian, 1 – 20133 Milan, Italy,* [2]*Institute of Cancer Research, Division of Epidemiology, Clifton Avenue, Sutton, SM2 5PX, UK.,* [3]*Institute of Occupational Health, University of Turin, Via Zuretti, 29 – 10126 Turin, Italy,* [4]*"Mario Negri" Institute, Via Eritrea 62 – 20157 – Milan, Italy*

**Summary** The effects of various factors were evaluated on both relative risk (multiplicative model), and absolute excess risk (additive model) of bladder cancer among 664 workers of a dyestuff factory in Northern Italy. These workers were exposed to aromatic amines in fairly constant working conditions from 1922 to 1970, and were employed for at least one year. They were followed up till the end of 1981 for a total of 12,302 man-years at risk. Under both models, the risk was greater for workers directly involved in aromatic amine manufacture than for those with only intermittent exposure. There was no marked effect of age at first exposure on the absolute excess risk of bladder cancer, but the relative risk was strongly and negatively related to age at first exposure. Under the multistage theory of carcinogenesis, this pattern of risk indicates an early stage effect. Absolute excess risk increased sharply during exposure, and continued to rise, although less sharply, after exposure had ceased. Relative risk, however, decreased after cessation of exposure, indicating a possible late stage effect. Thus, the results derived from both additive and multiplicative models are not in contrast when interpreted in terms of the multistage theory of carcinogenesis, though they are not totally consistent with a single-stage effect, either early or late. Aromatic amines may act on a stage somewhere between the first and penultimate, or on more than one stage of the process of carcinogenesis. Alternatively, it is possible that imprecision in the job classification or other observational problems may obscure the trends, or produce fictitious trends in the effects of variables such as age at first exposure and time since last exposure. Finally, such a pattern of trends could emerge if there were only two stages and the first and penultimate stage were the same.

Though the risk of bladder cancer for employment in the aromatic amine industry was reported as early as 1898 (Matanosky & Elliott, 1981), their production continued for several decades thereafter. Workers of a dyestuff factory in the district of Turin (Northern Italy) were exposed to naphthylamines, benzidine and other chemicals up to 1970, when a hospital based study showed 23 cases of malignant neoplasms of the bladder among these workers (Rubino & Coscia, 1973). Mortality of the whole cohort of workers employed in this factory between 1922 and 1970 ($n=906$) was followed up thereafter. A descriptive epidemiological paper has been previously published, showing an approximately 30-fold increased mortality for bladder cancer (based on 36 deaths) and a 50% elevated death rate for all causes (Rubino *et al.*, 1982). Furthermore, those data showed a markedly increased risk (more than 60-fold, based on 5 deaths) in workers involved in production of fuchsin and safranine T.

Correspondence: A. Decarli
Received 26 November 1984.

The information available on these workers has recently been updated to the end of 1981 to obtain a total of 16,222 man years at risk (12,302 in workers exposed to aromatic amines), and 41 bladder cancer deaths. Thus, the data of this cohort provide one of the few examples of human carcinogenesis where the number of excess cases is large enough to permit evaluation of the effects of age, duration of exposure and time since last exposure under various models of carcinogenesis.

### Subjects and methods

### *Characteristics of the study cohort*

The cohort comprised all men who had worked for at least one year in the factory between 1922 and 1970. Among these men (919), 10 who had died and 3 who had not been traced before 1946 were not considered, thus leaving a total 906 subjects under study.

Date of birth, of engagement and termination of employment, job particulars including categories of exposure to selected chemicals, and the last known

© The Macmillan Press Ltd., 1985

address were registered from personnel records at the factory. If death had been notified, death certificates were obtained from registration offices at place of death. Further verification of vital status was obtained from registries of current residence.

For the present report, follow-up was updated to December, 1981, giving a total number of 284 deaths (41 from bladder cancer), and 16,222 man-years at risk (12,302 among exposed subjects). Exclusion of untraced subjects (38; 4.2%) from computation of man-years at risk is unlikely to have materially influenced any of the results, as this accounted for 130/12,302 (1.1%) man-years among exposed subjects.

*Data analysis and models of risk*

All the analyses presented refer to the period 1946–81. The expected number of deaths from bladder cancer and all other causes (Table I) were computed using national mortality rates for each five year calendar period and age group (ISTAT, 1955–83). All-cause national mortality rates per each quinquennium of age are published in Italy by the Central Institute of Statistics (ISTAT) from 1951 onwards. Likewise, bladder cancer mortality rates were available only from 1951 onwards (elaborations from ISTAT unpublished data). Thus, 1951 rates were applied to the period 1946–51.

**Table I** Mortality experience for bladder cancer and all causes of 868[a] workers of a dyestuff factory in Northern Italy according to specified variables. Expected (Exp) numbers are based on national mortality rates in each five-year calendar period and age group. Deaths and man-years beyond age 85 are excluded.

| | *Bladder cancer* | | | *All other causes* | | | |
|---|---|---|---|---|---|---|---|
| *Variables* | *OBS* | *EXP* | *O/E* | *OBS* | *EXP* | *O/E* | *(Man-years/ no of subjects)* |
| *Period first employed* | | | | | | | |
| 1922–39 | 10 | 0.21 | 47.62 | 40 | 26.69 | 1.50 | (1,461/70) |
| 1940–54 | 24 | 0.53 | 45.28 | 118 | 66.78 | 1.77 | (7,120/310) |
| 1955–78 | 7 | 0.15 | 46.67 | 36 | 19.10 | 1.88 | (3,721/284) |
| *Age at first exposure* | | | | | | | |
| <25 | 8 | 0.04 | 200.00 | 24 | 10.27 | 2.34 | (3,645/166) |
| 25–34 | 12 | 0.19 | 63.16 | 43 | 24.43 | 1.76 | (4,153/207) |
| ⩾35 | 21 | 0.66 | 31.82 | 127 | 77.86 | 1.63 | (4,504/291) |
| *Duration of exposure* | | | | | | | |
| <5 | 6 | 0.24 | 25.00 | 69 | 30.46 | 2.27 | (4,442/299) |
| 5–9 | 9 | 0.19 | 47.37 | 49 | 24.73 | 1.98 | (2,898/157) |
| ≧10 | 26 | 0.46 | 56.52 | 76 | 57.37 | 1.32 | (4,962/208) |
| *Job category* | | | | | | | |
| A. αβ naphthylamine or benzidine manufacture | 27 | 0.19 | 142.11 | 35 | 22.04 | 1.46 | (2,563/151) |
| B. Naphthylamine or benzidine use | 3 | 0.18 | 16.67 | 45 | 22.91 | 1.96 | (2,616/147) |
| C. Intermittent contact with naphthylamines or benzidine | 6 | 0.46 | 13.04 | 96 | 58.88 | 1.63 | (6,167/312) |
| D. Fuchsin or safranine T manufacture | 5 | 0.07 | 71.43 | 18 | 8.75 | 2.06 | (956/54) |
| E. Other jobs not involving exposure or unknown[b] | 0 | 0.30 | — | 49 | 37.44 | 1.31 | (3,920/204) |
| *Time since last exposure* | | | | | | | |
| During exposure | 16 | 0.14 | 114.29 | 73 | 24.46 | 2.98 | (4,392/90) |
| <5 | 5 | 0.16 | 31.25 | 19 | 21.84 | 0.87 | (2,822/24) |
| 5–9 | 7 | 0.20 | 35.00 | 37 | 23.80 | 1.55 | (2,277/180) |
| ⩾10 | 13 | 0.39 | 33.33 | 65 | 42.50 | 1.53 | (2,811/370) |
| TOTAL | 41 | 0.89 | 46.07 | 194 | 112.60 | 1.72 | (12,302/664) |

[a]38 untraced subjects excluded.
[b]These workers were excluded from any other analysis, including the other variables and total in **Table I**.

Only the date of death and not the actual date of diagnosis was available to us. As retirement or change of job may in some cases have been due to the disease itself, deaths that occured within three years of stopping exposure were considered together with those during exposure. Changing this interval to two or four years did not materially change any of the results.

Two general models (Breslow *et al.*, 1983) were considered for the cancer death rates in workers exposed to aromatic amines. These were, first, the multiplicative (or relative risk) model

$$O_{(k)} = E_{(k)} e^{\beta' Z(k)} \quad (1)$$

where $O_{(k)}$ is the observed number of bladder cancer deaths in the $K^{th}$ category, $E_{(k)}$ is the expected number of deaths in the $K^{th}$ category based on national mortality rates, $Z_{(k)}$ is a vector of covariates (such as age at first exposure, job etc.) whose influence on the event is being examined, and $\beta'$ is a vector of unknown parameters, to be estimated. In this model, the effect of each factor acts multiplicatively on the expected rate in the general population.

The second general model considered was the additive (or excess risk) model

$$O_{(k)} = E_{(k)} + MY_{(k)} \times e^{\beta' Z(k)} \quad (2)$$

with variables defined as above, and $MY_{(k)}$ defined as the total number of man-years in the category $K$. In this model, each factor is assumed to act multiplicatively on the excess risk, and the resulting product then adds to the expected number in the general population.

In general, as pointed out by Brown & Chu (1983), only the absolute excess risk measure discriminates quantitatively between early and late stage carcinogens when examining excess risk patterns while exposure continues. However, since the main feature of this cohort is the possibility of considering also the effect of cessation exposure, it is useful to examine models for both relative and excess risk, to provide a full description of the relationship between exposure factors and cancer under study, and bearing in mind that models (1) and (2) can both be interpreted under the multistage theory of carcinogenesis.

Under model (2), the excess risk is constrained to be positive (although of course it can be arbitrarily small), so the model cannot be used to represent a protective effect. However, theoretically, this appears to be a reasonable restriction, since a protective effect would be expected to act multiplicatively on an existing cancer risk. Furthermore, in the present work, the observed mortality is so much greater than the expected one that this restriction would not appear to have any practical implication.

Equations (1) and (2) can be easily fitted using GLIM (Baker & Nelder, 1978) with appropriate user supplied macros (Kaldor *et al.*, in press). The interpretation of the results under both models is, in fact, assimilable to a log-linear model on a multi-way contingency table.

### Results

Table I shows observed and expected numbers of deaths from bladder cancer and all other causes according to variables of interest. Bladder cancer mortality did not vary markedly with calendar period of first exposure: in fact, there were no substantial changes in manufacturing techniques until about 1970. Thus, this variable was not considered in any further analysis. The crude observed/expected ratio for bladder cancer deaths was greater for workers who had started working before age 25, and decreased with increasing age at first exposure. Likewise, the risk increased with increasing duration of exposure and, clearly, was greater for workers directly involved in aromatic amine manufacture than for those with only intermittent exposure. No death from bladder cancer up to age 85 was observed in workers employed in jobs not involving exposure to aromatic amines (i.e., finished product workers etc., though one death from bladder cancer was observed over age 85, *vs* 0.3 expected). Thus, these workers were excluded from any further analysis. The increased risk was apparently larger (O/E = 114.3) during exposure, but no clear trend of decreased risk with increasing time since last exposure was evident, the observed/expected ratio remaining around a factor 30 more than ten years after cessation of exposure.

Table II reports the deviances obtained fitting all combinations of the variables age at first exposure (AF) duration of exposure (D), job category (J), and time since last exposure (TL) to the bladder cancer deaths under the multiplicative (1) and the additive model (2), without interaction terms. The deviance can be interpreted as a goodness-of-fit statistic, with smaller values implying a closer fit to the model.

Under both models, the variable J produced the largest reduction in deviance, either by itself or in combination with other variables.

Other variables, however, had different effects under the two models. Under the multiplicative model (1), AF, together with J, explained most of the fitting, though a significant reduction was given

**Table II** Deviance obtained for combination of variables Age at first exposure (AF), Duration of exposure (D), Job category (J) and Time since last exposure (TL) fitted to the bladder cancer deaths.

| *Variables* | *Degrees of freedom* | *Multiplicative model (1)* | *Additive model (2)* |
|---|---|---|---|
| AF | 332 | 178.0 | 206.5 |
| D | 332 | 186.5 | 185.5 |
| J | 331 | 146.6 | 163.6 |
| TL | 331 | 184.0 | 208.4 |
| AF+D | 330 | 175.5 | 180.9 |
| AF+TL | 329 | 170.3 | 204.4 |
| AF+J | 329 | 129.0 | 162.1 |
| D+TL | 329 | 179.9 | 182.1 |
| TL+J | 328 | 138.7 | 160.9 |
| D+J | 329 | 139.4 | 135.0 |
| AF+D+TL | 327 | 168.9 | 177.1 |
| AF+D+J | 327 | 125.6 | 132.6 |
| AF+TL+J | 326 | 120.7 | 159.1 |
| D+TL+J | 326 | 132.9 | 131.3 |
| AF+D+TL+J | 324 | 118.7 | 128.5 |

by TL too, whereas D made no significant contribution in the presence of AF and J. Under the additive model (2), on the other hand, a large contribution was made by D, whereas the reduction in deviance due to AF and TL was not significant in the presence of J and D.

The fit was generally satisfactory for both models, being somehow closer for the multiplicative model for most combinations. However, for a number of combinations the difference was not large, and for a few combinations the additive model fitted slightly better. However, there is no formal way to test the significance of the difference between the fit of the two models at any combination of variables, since the models are not nested.

Table III reports the parameter estimates obtained when all four variables were fitted to the bladder cancer deaths, under models (1) and (2). In addition to the parameter estimates and their estimated standard errors, the estimates for each variable are expressed in relation to one of the categories of the variable, arbitrarily chosen as the baseline.

A test for the significance of each parameter can be made by comparing the ratio of the parameter to its standard error to a standard normal deviate.

Both relative risk and excess risk were closely related to job category, with fairly similar estimates. For the other three variables considered, however, there were clear distintions between the two models. Relative risk dropped strongly with age at first exposure, whereas excess risk showed no clear pattern or, if any, an elevated risk in subjects aged 35 or older when starting work. No clear trend of increasing relative risk with longer exposure was evident, though the risk estimates were (not

**Table III** Parameter estimates obtained by fitting Age at first exposure (AF), Duration of exposure (D), Job category (J) and Time since last exposure (TL) to bladder cancer risk

| | | *Multiplicative model (1)* | | | *Additive model (2)* | | |
|---|---|---|---|---|---|---|---|
| *Variable* | *Level* | *Parameter* | *(SE)* | *Relative risk* | *Parameter* | *(SE)* | *Absolute excess risk* |
| AF | <25 | = | = | 1.00 | = | = | 1.00 |
| | 25–34 | −0.87 | (0.46) | 0.42 | 0.05 | (0.29) | 1.05 |
| | ⩾35 | −1.74 | (0.45) | 0.17 | 0.57 | (0.27) | 1.77 |
| D | <5 | = | = | 1.00 | = | = | 1.00 |
| | 5–9 | 0.67 | (0.53) | 1.94 | 1.34 | (0.34) | 3.84 |
| | ⩾10 | 0.59 | (0.48) | 1.80 | 2.24 | (0.29) | 9.40 |
| J[+] | A | = | = | 1.00 | = | = | 1.00 |
| | B | −2.37 | (0.60) | 0.09 | −2.34 | (0.40) | 0.09 |
| | C | −2.52 | (0.45) | 0.08 | −2.59 | (0.30) | 0.07 |
| | D | −0.88 | (0.49) | 0.41 | −0.58 | (0.31) | 0.56 |
| TL | During Exp. | = | = | 1.00 | = | = | 1.00 |
| | <5 | −0.52 | (0.45) | 0.59 | −0.05 | (0.29) | 0.95 |
| | 5–9 | −0.94 | (0.47) | 0.39 | 0.14 | (0.30) | 1.16 |
| | ⩾10 | −1.01 | (0.40) | 0.36 | 0.76 | (0.25) | 2.14 |

[a]See **Table I** for definition.

significantly) above unity; excess risk, on the other hand, increased strongly with longer exposure. Conversely, relative risk dropped with time since last exposure, whereas excess risk was significantly larger in subjects who had stopped working ten years before.

### Discussion

We have examined the effects of various factors on both relative risk and absolute excess risk of bladder cancer among workers at a dyestuff factory in Northern Italy exposed to aromatic amines in fairly constant working conditions from 1922 to 1970. Both relative risk and excess risk were clearly greater for workers directly involved in naphthylamine and benzidine and, (to a lesser extent, relative risk estimates being half that for naphthylamine and benzidine) fuchsin and safranine T manufacture, than for those with only intermittent exposure.

When adjusted for duration and time since last exposure, the excess bladder cancer produced by aromatic amine exposure did not show a strong dependence on age at which workers had begun employment, though the excess risk was slightly elevated in those starting working at age 35 or older. This apparent effect of age at full exposure was more evident in men who were still employed when they died (though this interaction on a small series may be due to chance).

Under the multistage theory of carcinogenesis (Armitage & Doll, 1961; Day & Brown, 1980), independence of the excess risk of age at first exposure would suggest an effect on one of the early stages. The relative risk, however, in the case of an early stage effect would be a decreasing function of age at first exposure: this was exactly what was borne out by the multiplicative model.

Thus, the results derived both from model (1) and (2) are roughly consistent with the predictions of the multistage theory of carcinogenesis, if the effect of aromatic amine exposure was on one of the early stages. The effect of duration explains most of the excess risk under the additive model (2). This is consistent with an early stage effect, too. Under the multiplicative model, however, duration had only a moderate (and non significant) effect when allowance was made for age at first and time since last exposure. This is however, not surprising, since the absolute excess risk is an increasing function of duration, $f(d)$, whereas the relative risk is a function of $(d/t)$ where $t$ is age (thus the sum of age at first exposure, duration, and time since last exposure) (Brown & Chu, 1983).

Absolute excess risk continued to rise up to ten or more years after cessation of exposure. Relative risk, however, decreased after cessation of exposure, this indicates, under the multistage theory, a late stage effect.

In this regard, too models (1) and (2) are, therefore, not in contrast when interpreted in terms of the multistage theory of carcinogenesis. However, the finding that the excess risk is simultaneously an increasing function and the relative risk a decreasing function both of AF and of TL is not consistent with a single-stage effect, either early or late. It is still possible that aromatic amines act on a stage somewhere between the first and penultimate one, or on more than one stage of the process of carcinogenesis. Alternatively, such a pattern of trends could emerge if there were only two stages, the first and penultimate stage being the same. However, there are obvious difficulties in such interpretations of occupational exposure data in the context of the multistage theory. Although the duration of exposure has been formally controlled for in the model, it may still have a confounding effect if its relation to the relative or excess risk is not of the simple multiplicative form specified by models (1) or (2). Even if the model is finally correct, the imprecision of the job classification as a measure of exposure may obscure the trends, or produce fictitious trends in the effects of variables such as age at first exposure and time since last exposure.

From a purely numerical standpoint, both the multiplicative and the additive models appear to fit the data satisfactorily, and both models can be interpreted (though with some difficulties) under the multistage theory of carcinogenesis. Therefore, there appears to be no obvious choice between the multiplicative and the additive model.

Bladder cancer is related to cigarette smoking, (Surgeon General, 1982), and the cohort under study showed an elevated risk (compared to national mortality rates) of several smoking related cancers: lung (observed/expected ratio = 1.8), larynx (O/E = 3.6), and oesophagus (O/E = 4.1) (Rubino *et al.*, 1982).

In this respect, the definition whether the effect of aromatic amine exposure and cigarette smoking on bladder cancer risk is additive rather than multiplicative has important implications for the calculation of lifetime risk estimates (Kaldor *et al.*, in press). In general, under the additive model the background bladder cancer rate has little effect on the lifetime risk estimates, whereas under the multiplicative model the individual's background rate substantially effects the estimate. Table IV presents cumulative probabilities of bladder cancer death for a worker born in 1905 who strated working at the dyestuff factory in 1935 and worked

**Table IV** Cumulative probabilities[a] of bladder cancer death for the average male born in Italy in 1905, and for a male born in 1905 who worked for ten years at the dyestuff factory starting in 1935

| | *Cumulative probabilities (%) ignoring competing risk* | |
|---|---|---|
| | *Multiplicative model (1)* | *Additive model (2)* |
| Average male | 0.77 | |
| Dyestuff worker according to job category[b] | | |
| A | 89.97 | 39.26 |
| B | 14.27 | 4.94 |
| C | 12.77 | 4.03 |
| D | 53.28 | 24.32 |
| Dyestuff worker according to cigarette smoking[c] | | |
| Non smoker | 18.81 | 13.17 |
| Average smoker | 34.90 | 13.51 |
| Heavy smoker | 60.04 | 14.20 |

[a]Up to age 75 yr, in the absence of other causes of death.
[b]See **Table I** for definition.
[c]The models account for AF, D, TL effects only.

there for ten years, according to job category and smoking level, assuming that a non-smoker had half and a heavy-smoker twice the risk of the average Italian male.

We thank Professor G. Rubino, Director of the Institute of Occupational Health, University of Turin, for allowing us to analyse data collected in his Institute; Dr. J. Kaldor, International Agency for Research on Cancer, Lyon, for valuable advice and assistance in data analysis; Ms. Carol Hermon, ICRF Cancer Epidemiology and Clinical Trials Unit, Oxford for help with computer programming; Ms. Angela Simm and Nadia Triulzi for editorial assistance.

This work was partly supported by C.N.R. (Italian National Research Council) grants No 82.02038.56, 82.02045.56 and 84.00639.44 within the framework of the Applied Projects "Preventive and Rehavilitative Medicine" and "Oncology". The work of Prof. Decarli was partly conducted under the terms of a travel fellowship provided by the Italian Association for Cancer Research.

### References

ARMITAGE, P. & DOLL, R. (1961). Stochastic models for carcinogenesis. In: *Proceedings of the Fourth Berkeley Symposium on Mathematical Statistics and Probability.* Vol. 4, Berkeley, p. 19 Ed. Neyman, University Press, California.

BAKER, R.J. & NELDER, J.A. (1978). *Generalized Linear Interactive Modeling (GLIM) System.* Release 3. Oxford: Numerical Algorithms Group.

BRESLOW, N.E., LUBIN, J.H., MAREK, P. & LANGHOLZ, B. (1983). Multiplicative models and cohort analysis. *J. Am. Stat. Ass.*, **78**, 1.

BROWN, C.C. & CHU, K.C. (1983). A new method for the analysis of cohort studies: Implications of the multistage theory of carcinogenesis applied to occupation arsenic exposure. *Environ. Health Perspect.*, **50**, 293.

DAY, N.E. & BROWN, C.C. (1980). Multistage models and primary prevention of cancer. *JNCI*, **64**, 977.

ISTAT. Annuario Statistico Italiano, Vol. I–XXIV, 1955–1979, Roma, 1958–1983.

KALDOR, J. PETO, J., DAY, N., DOLL, R., HERMON, C. & MORGAN, L. Models for respiratory cancer in nickel workers. (In press)

MATANOSKI, G.M. & ELLIOTT, E.A. (1981). Bladder Cancer Epidemiology. *Epidemiol Rev.*, **3**, 203.

RUBINO, G. & COSCIA, G.C. (1973). I tumori professionali del tratto urinario. *Il Cancro*, **3**, 151.

RUBINO, G.F., SCANSETTI, G., PIOLATTO, G. & PIRA, E. (1982). The carcinogenic effect or aromatic amines: An epidemiological study on the role of o-toluidine and 4,4′-methylene bis (2-methylaniline) inducing bladder cancer in man. *Environ. Res.*, **27**, 241.

SURGEON GENERAL (1982). *The Health Consequences of Smoking: Cancer.* Washington: U.S. Government Printing Office.